# Advances in retinal imaging biomarkers for the diagnosis of cerebrovascular disease

**DOI:** 10.3389/fneur.2024.1393899

**Published:** 2024-09-19

**Authors:** Yier Zhang, Ting Zhao, Ling Ye, Sicheng Yan, Wuyue Shentu, Qilun Lai, Song Qiao

**Affiliations:** ^1^The Second Clinical Medical College, Zhejiang Chinese Medicine University, Hangzhou, Zhejiang, China; ^2^Department of Neurology, Zhejiang Hospital, Hangzhou, Zhejiang, China; ^3^Department of Geriatrics, Jinhua Fifth Hospital, Jinhua, Zhejiang, China

**Keywords:** cerebrovascular disease, retina, biomarkers, cerebral microcirculation, brain function

## Abstract

The increasing incidence and mortality rates of cerebrovascular disease impose a heavy burden on both patients and society. Retinal imaging techniques, such as fundus photography, optical coherence tomography, and optical coherence tomography angiography, can be used for rapid, non-invasive evaluation of cerebral microcirculation and brain function since the retina and the central nervous system share similar embryonic origin characteristics and physiological features. This article aimed to review retinal imaging biomarkers related to cerebrovascular diseases and their applications in cerebrovascular diseases (stroke, cerebral small vessel disease [CSVD], and vascular cognitive impairment [VCI]), thus providing reference for early diagnosis and prevention of cerebrovascular diseases.

## 1 Introduction

**Figure 1:**
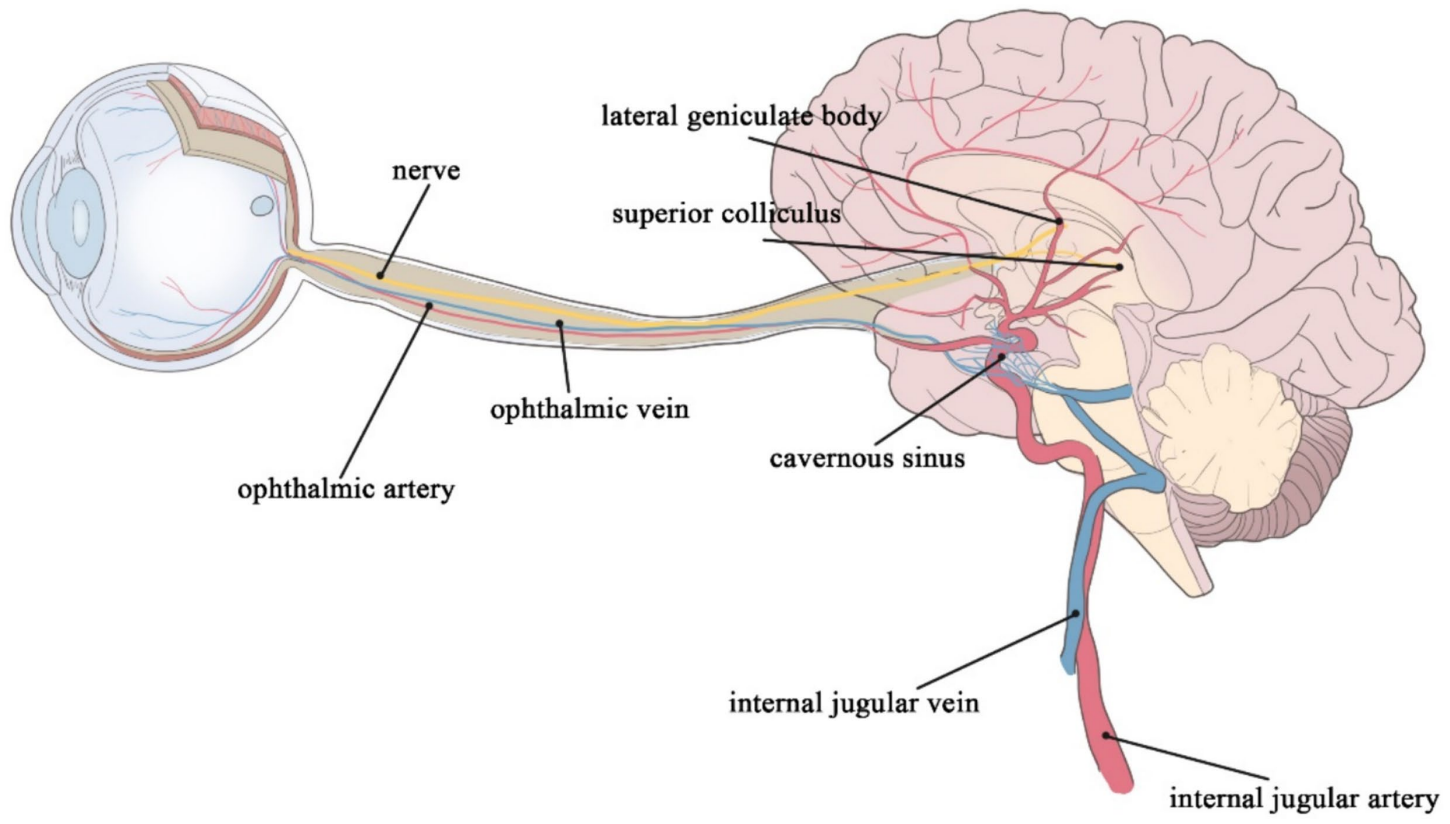
The retinal artery merges into the internal carotid artery; the retinal vein merges into the cavernous sinus and finally into the internal jugular vein; the ophthalmic nerves continue posteriorly as the optic tract, which wraps around the cerebral peduncle to the lateral geniculate body of the thalamus; before it reaches the lateral geniculate body, the tract divides into a lateral heel and a medial root, most of which passes through the lateral root to the lateral geniculate body, while optic tract fibers of the medial root pass below the medial geniculate body to the superior colliculus of the midbrain.

Cerebrovascular lesions in patients with cerebrovascular disease (CVD) promote cerebral dysfunction. CVD includes limited or diffuse cerebral dysfunction elicited by various cerebrovascular lesions, such as vascular lumen occlusion or stenosis, vascular rupture, vascular malformation, vascular wall injury, or altered permeability, but excludes diffuse cerebral dysfunction caused by hemodynamic abnormalities and other factors leading to total cerebral ischemia or hypoxia. CVD diagnosis presently relies on imaging technology. Nevertheless, conventional imaging technologies cannot efficiently detect the composition of small blood vessels and lesions within the brain. In addition, conventional imaging technologies can be invasive and expensive, thus unsuitable for the initial screening of cerebrovascular disease in the general population. Moreover, imaging findings reflect indirect signs of vascular changes in the brain. Early cerebrovascular lesions can precede alterations detected by imaging studies, such as magnetic resonance imaging (MRI) (1). In-depth research on CVD pathogenesis has highlighted various types of pathogenesis, yielding several detectable markers. The retinal system and the brain system share similar embryonic origins, anatomical features, and physiological properties. The retina and optic nerve extend from the diencephalon, thus considered part of the central nervous system (Figure 1) (2, 3). Notably, both systems have similar barrier and regulatory functions. Lesions in the retinal vasculature can reflect changes in cerebral vessels, such as fundus microangiomas, microhemorrhages, and possible associations with stroke and cerebral small vessel disease (CSVD) (4). Therefore, retinal signs and lesions may be crucial for investigating CVD (2). Non-invasive observation of retinal vascular changes and the early detection of these changes may reveal related cerebral pathologies, showing promise as a novel target for the screening and prevention of CVD (5).

Initially, fundus imaging methods mainly relied on fundus imaging infrared (IR), indocyanine green angiography (IGGA), fluorescein fundus angiography (FFA), etc. Fundus imaging uses lasers, injection of fluorescent dyes, etc., to observe the structure of the retina and vascular morphology. Optical coherence tomography (OCT) technology appeared at the beginning of the 21st century and has been used to generate high-resolution images, non-invasive observation, and rapid access to the various structures of the retina. However, OCT cannot be used to observe the vascular situation (6). Currently, optical coherence tomography angiography (OCTA) is widely used to observe the dynamics of the blood vessels in the fundus without the need to inject a dye. Swept-source optical coherence tomography (SS-OCT) has a longer and deeper wavelength, enabling a wider range of access to the various structures of the retina. The improvement of OCT-related technologies can achieve faster imaging and higher resolution, while reducing the appearance of pseudovessels in deep imaging (7). In addition, multimodal imaging and holographic fundus imaging are new developments in the field of retinal imaging technology. Multimodality can combine different imaging modalities to provide more comprehensive retinal information, while holography can acquire fundus images with a wider field of view and high resolution.

Retinal biomarkers of cerebrovascular lesions can be classified into three primary categories: static vascular morphology of the retina, dynamic blood flow conditions in the retina, and tissues other than the retinal vasculature. Static vascular morphology of the retina, such as vessel diameter, vessel density (VD), vessel wall thickness, fractal dimension, arteriovenous diameter ratio, vascular tortuosity, etc. (8–12), can be measured via fundus photography, FFA, and OCTA, etc. Dynamic blood flow conditions in the retina, including blood flow velocity, resistance index, pulsatility index, and fluctuations in vessel diameter after flash stimulation (13–16), can be determined via instrumental methods, such as laser speckle flow imaging system, laser scanning Doppler flowmetry, and retinal oximetry, etc. Other tissues other than the retinal vasculature, such as macular thickness and volume changes, retinal nerve fiber layer (RNFL) thickness, and macular foveal avascular zone (FAZ) (17–20), can be measured through OCT, OCTA, and SS-OCT.

## 2 Application in stroke patients

### 2.1 Relationship with ischemic stroke

Stroke is divided into two: ischemic stroke (IS) (80%) and hemorrhagic stroke (HS). This review also focuses on retinal imaging markers associated with IS patients. Many scholars and institutions have evaluated retinal vascular-related imaging markers. In Doubal et al.’s study, retinal microvascular and neural alterations in IS patients were detected using OCTA, and showed that the occurrence and staging of lacunar strokes are associated with a decrease in the fractal dimension (FD) (21). Their result shows the low FD is associated with a 2-fold higher risk of stroke events compared with high FD (stroke incidence: 42.3% vs. 27.8%; OR 2.30, 95% CI 1.06–4.97). This connects with the study by Kawasaki et al. (22). They also showed that the microvascular network in the retina of patients with ischemic stroke is sparser, more tortuous, and has a lower fractal dimension, which doubles the risk of stroke occurrence. Fractal dimension is a measure of the geometry of retinal blood vessels (small arteries and veins). A decrease in retinal FD represents sparse branching of retinal blood vessels, possibly due to vascular atrophy and collapse caused by hypoxia. In contrast, a decrease in blood oxygen concentration in the brain can lead to chronic cerebral ischemia by causing various pathological changes, such as arterial spasm, smaller vessel caliber, atherosclerosis, and brittleness of blood vessels. Therefore, FD of the eye can be used to assess cerebral ischemic status.

Seidelmann et al. conducted a long-term follow-up trial with the endpoint indicator of whether IS, coronary heart disease (CHD), heart failure (HF), and fatal events occurred after the third visit to assess the vascular morphologic aspects of the retina. The final results showed that a narrower caliber of small retinal arteries and a wider caliber of small veins are significantly associated with ischemic stroke (*p* < 0.001) (23). However, several cohort studies, such as the Blue Mountains Eye Study (24, 25), the Beaver Dam Eye Study (25), and the Rotterdam Study (26), have described that the incidence of stroke and mortality are associated with an increase in the caliber of the small retinal veins but not with a change in the caliber of the small retinal arteries. This may be because caliber of retinal arterioles is susceptible to hypertension, diabetes, and other factors, thus subject to dynamic blood flow, which may be altered during the time point of still image capture (27). Ong et al. found a correlation between changes in the caliber of the retinal small arterioles and the occurrence of stroke. Ong et al. also found an independent correlation between increased small arteriolar curvature and the occurrence of stroke, suggesting that this indicator is superior to changes in arteriolar caliber (28). Vascular tortuosity is the severe twisting of blood vessels caused by an increase in vessel length, possibly due to insufficient arterial blood supply, damage, or sclerosis of the vessel wall. Increased tortuosity of small retinal arterioles indicates inadequate blood supply to retinal blood flow and vessel wall dysfunction. Vascular tortuosity is more accurate than changes in vessel caliber because it is relatively static and has less temporal deformability. A recent study assessed vascular parameters and changes in the avascular zone of the central retinal recess in IS patients and found that higher FAZ axis ratio (FAR) (OR = 2.77, *p* = 0.008) and lower FAZ circularity (FC) (OR = 0.36, *p* = 0.022) in the deep capillary plexus (DCP) of the retina are associated with the occurrence of stroke (29). Few studies have investigated changes in the shape of the FAZ. Most of these studies have used the index of FAZ area because it is easier to measure and obtain. Previous studies have found that FAZ area is increased in patients with stroke (30–33). In contrast, this study used a morphologic alteration index of the FAZ and found that increased irregularity of the avascular zone of the deep capillary plexus in patients with total IS is correlated with stroke. The deep capillary plexus is located at the junction of the inner and outer plexiform layers and has a low oxygen content. This further aggravates the hypoxic condition of the retinal vasculature after stroke and the accumulation of metabolites, such as lactic acid and adenosine, resulting in the dilatation and deformity of the vasculature.

The correlation between other metrics, such as RNFL thickness and stroke occurrence has also been studied. Ye et al. collected retinal parameters via a multimodal study and assessed their relationship with multimodal functional connectivity and visual performance of the brain in 46 patients with thalamic stroke. Results showed that thickness of the retina (ß = −3.927, *p* = 0.008) and superficial vascular complex (SVC) (ß = −10.979, *p* = 0.013) are correlated with functional connectivity in subgroups of patients with a duration of ≤6 months and >6 months (34). Similar results were reported in another paper examining thalamic stroke and retinal structural changes. The results found that peripapillary retinal nerve fiber layer (pRNFL) thickness is also correlated with stroke, with thinner peripapillary retinal nerve fibers in stroke patients almost similar to that of healthy controls (35). This may be related to structural changes in the retina due to lesions in the visual pathway and subsequent retrograde degeneration after thalamic stroke. Several other studies have also found that RNFL thinning may predict lacunar stroke (8, 36). Other signs of retinal microvascular abnormalities, such as arteriovenous cuts, reduced arteriovenous ratio (AVR), and retinal microaneurysms, are possible markers of stroke events (37–39). These findings provide additional marker references for early diagnosis and assessment of stroke occurrence.

### 2.2 Relationship with stroke subtypes

Retinal microvascular signs may vary depending on stroke type (Toast). There are recent studies using OCTA images to identify ischemic stroke and its subtypes. Xiong et al. found superficial vascular plexus (SVP) yielded the highest areas under the receiver operating characteristic curve (AUCs) of 0.922 and 0.871 for the ischemic stroke detection and stroke subtypes classification (40). This has good predictive value. When parameterizing the OCTA images, they showed individuals with ischemic strokes had increased SVP tortuosity (ß = 0.085, 95%, confidence interval [CI] 0.005–0.166, *p* = 0.038) and reduced FAZ circularity (ß = −0.212, 95% CI −0.42 to −0.005, *p* = 0.045). This led them have the conclusion that SVP angiograms has the highest discriminatory power to detect microvascular changes between patients with ischemic stroke and healthy controls. The SVP is a network of both large and small vessels connected directly to the retinal arteries and veins and supplies all other vascular plexuses. Morphological changes in SVP may be sensitive to the structural changes that occur in ischemic stroke and may be caused by downstream effects of cerebral microcirculatory pathology. Therefore, it can be argued that retinal vascular changes have the potential to be used as a screening tool to detect microvascular changes in patients with ischemic stroke. Besides, studies have shown that retinal microvascular parameters vary among stroke patients according to toast type. Carotid arteries are the large arteries with the highest cerebral relevance. Carotid artery atherosclerosis or stenosis is a major risk factor for stroke. Carotid artery stenosis can lead to decreased blood flow to the arteries of the eye and a range of pathologies. Zhao et al. showed that patients with large-artery atherosclerotic strokes have the widest mean microvessel diameters with mean vein diameters being >82.23 μm (range; 0.5–1.0 optic disc diameters). This result can predict the development of large atherosclerotic stroke (95% CI 0.566–0.815, *p* = 0.021) (41). Rhee et al. also showed that patients with carotid artery stenosis have wider central retinal vein diameters than normal controls. They also showed that a reduced diameter of the central retinal artery (CRA) is also associated with stroke development (42). Studies have shown that a wider caliber of small retinal veins and a narrower caliber of small arteries are markers of endothelial dysfunction and inflammation, as well as indicators of impaired cerebral oxygen perfusion (43). In addition, Kwapong et al. studied the relationship between retinal microvascular parameters and cerebral perfusion parameters in 37 patients with carotid artery stenosis and showed that the RNFL, ganglion cell-inner plexiform layer (GCIPL) are significantly thinner in eyes ipsilateral to the stenosis. They also showed that the relative cerebral blood volume (rCBV) and the relative cerebral permeability surface (rPS) are associated with RNFL (*p* < 0.001) and GCIPL (*p* < 0.001) (44). Permeability surface (PS) can be used to indirectly measure blood–brain barrier (BBB) permeability, whereas cerebral blood volume (CBV) describes the volume of blood in cerebral capillaries and small veins per brain tissue volume. The RNFL and GCIPL are the components of the inner blood-retinal barrier (iBRB) (45), forming the SVC containing the small arterioles, which is the main blood flow pathway into the retina. Therefore, the significant correlation between rPS, rCBV, and retinal structural thickness suggests that changes in the brain may reflect changes in the retina and vice versa. Rita et al. (46) measured middle cerebral artery blood flow velocity and retinal vascular density using transcranial Doppler (TCD) and OCTA, respectively. They also assessed the correlation between the measurements, transient hyperemic response ratio (THRR), and cerebral arterial resistance transient hyperemic response ratio (CAR-THRR) with retinal vascular density. The results suggested that total retinal vascular density and macular vascular density are greatly reduced in patients with carotid artery stenosis. Besides, there was a significant negative correlation between CAR-THRR with VD peripapillary small vessels (VDPP_small_) and vessels (VDPP_all_). These findings suggest OCT/OCTA imaging may be a promising tool for reflecting cerebral hemodynamic changes in patients with carotid artery stenosis (CAS) or large atherosclerotic stroke.

In a recent study, JaeSeong et al. used fundus retinal photographs to develop a predictive model for screening and staging Moyamoya disease (MMD) (47). Their study found that the fundus vascular region had a high sensitivity (89.8%) and specificity (90.4%) for detecting MMD and predicting its staging. MMD is characterized by progressive stenosis and occlusion of the internal carotid artery, with the main site of manifestation near the ophthalmic artery. As a result, retinal vessels may also be affected, making measurement of these changes through the retinal vessels valuable for confirming the disease and determining its staging. Previously, magnetic resonance angiography (MRA) was the standard method for evaluating arterial stenosis. However, using retinal images for evaluation is safer and more readily available. Furthermore, Cheung et al. found that the arteriovenous crossover sign is only associated with the development of lacunar infarction (LI) but not with other stroke subtypes. They studied several retinal microvascular parameters and brain imaging data in 810 patients and showed that LI occurred in 131 cases (16.2%). Moreover, patients with arteriovenous cross-signs were 2.82-fold more likely to develop LI than those without arteriovenous cross-signs (OR = 2.48, 95% CI 1.39–4.40) (48). In contrast, Duan et al. showed that the vascular oriented distribution (VOD) of the superficial capillary plexus (SCP) is associated with lacunar cerebral infarction (29) by comparing 33 IS patients (14 non-lacunar cerebral infarction and 19 lacunar cerebral infarction) with 27 controls. They showed that the lower VOD of the SCP (OR = 0.41) was independently associated with lacunar cerebral infarction. Studies have shown that patients with lacunar stroke are more likely to have retinal microvascular signs, such as smaller AVR, arteriovenous crossing abnormalities, or enhanced small-artery light reflexes, than patients with other stroke subtypes (49).

Thinning of the RNFL, narrowing of the central retinal arteriole (CRAE), widening of the central retinal vein (CRVE), focal small-artery stenosis, and altered vascular curvature can also be used to predict lacunar stroke (8, 36, 38). However, these retinal imaging markers are not specific and do not correlate with other types of stroke. Stroke has no independently associated retinal imaging markers due to cardiogenic embolism. Besides, the index results were similar to those of the large atherosclerotic and small artery occlusion types. Therefore, further research should investigate the correlation and specificity between stroke subtypes and retinal imaging markers. Therefore, future studies should rely on some special retinal imaging markers to distinguish different subtypes of stroke and some common markers to predict the risk of developing stroke.

### 2.3 Relationship with stroke recurrence

IS survivors are at high risk of experiencing stroke recurrence within 1 year. About 80–85% of patients who survive a first IS have at least one recurrence, with 8–15% of recurrences occurring in the first year (50). Recurrent strokes are more severe, more difficult to treat, and have a higher mortality rate than first-time strokes. Therefore, the prevention of stroke recurrence is of great clinical value. Zhuo et al. found that the risk of stroke recurrence increases when the angle of branching of the small retinal arteries decreases, and AVR decreases in stroke patients. Therefore, they proposed a prediction model for the risk of stroke recurrence, which was highly accurate (sensitivity: 72.5%, specificity: 70.7%, overall accuracy: 64.2%) (51). A predictive model for stroke recurrence risk was developed by Zhao et al. in their recent study (52). They recruited 141 patients with acute ischemic stroke and conducted a prospective study. Retinal imaging was performed immediately after the patients were admitted to the hospital and followed up for 3 years. The study found that patients with a mean arterial diameter (MAD) of 0.5–1.0 disc diameters ≥74.14 μm and a mean venous diameter (MVD) of 0.5–1.0 disc diameters ≥83.91 μm were more likely to experience a recurrent cerebrovascular event. The researchers also established a multivariate Cox proportional risk regression model and found that including MAD and MVD in addition to traditional risk factors improved the predictive potential for subsequent cerebrovascular events. This suggests that changes in retinal small vein and artery diameter are independently associated with recurrent cerebrovascular events, and their inclusion in the model enhances the accuracy of predicting such events in patients with acute ischemic stroke. Therefore, traditional stroke and recurrence risk factors, in combination with retinal vascular characteristics, should be considered to improve the accuracy of cerebral infarction recurrence prediction and early preventive measures.

In addition, some studies have found that fundus vascular sclerotic lesions are associated with the risk of stroke recurrence due to the similarity between the fundus vasculature and the cerebral vasculature. Yang et al. found that the occurrence of enlarged perivascular space (ePVS) in the brain is related to the presence or absence of sclerosis in the diameter of the CRA and the degree of sclerosis. Specifically, patients with intracranial ePVS secondary to stroke often have sclerosis of retinal arteries (*p* < 0.05) (53). Zhuang et al. studied the association between the degree of fundus arteriosclerosis and retinal signs with secondary ePVS in 266 patients with mild acute cerebral infarction and found that the number of ePVS is associated with the degree of fundus arteriosclerosis (ß = 0.586, 95% CI 0.345–0.827, *p* = 0.016) and retinal arteriovenous crossover sign (ß = 0.472, 95% CI 0.291–0.653, *p* = 0.010), which are positively and negatively correlated with the retinal AVR (ß = −3.974, 95% CI −5.548 to −2.400, *p* = 0.012), respectively. These findings suggest that the smaller the diameter of the CRA and retinal AVR, the more severe the fundus vasculopathy and intracranial ePVS (54). ePVS is one of the risk factors for stroke recurrence. Studies have shown that retinal vasculopathy signs combined with stroke risk factors can be used to predict stroke recurrence. However, only a few studies have assessed the correlation between retinopathy and stroke recurrence. ALL the second block above is summarized in Table 1.

**Table 1:** Summary of stroke-related biomarkers for retinal imaging.

Stroke	Related retinal imaging markers	Relevance	Reference
Stroke occurrence	FD	FD decreased	(21, 22)
	Caliber of small retinal arteries	Narrower Caliber of small retinal arteries	(23–26)
	Vascular tortuosity	Caliber of small retinal veins enlarge	(28)
	FAZ	Vascular tortuosity increased	(29)
	RNFL	degree of irregularity of the FAZ increased	(29–33)
		RNFL decreased	(8, 34–36)
Different stroke types	SVP	SVP tortuosity increased	(40)
	Large-artery atherosclerotic strokes	Large-artery atherosclerotic strokes	(41)
	CRV	CRV increased	(42, 43)
	CRA	CRA decreased	(42, 43)
	VD	VD decreased	(46)
	RNFL, GCIPL	Thinner RNFL, GCIPL	(44, 45)
	Arteriovenous crossover sign	Arteriovenous crossover sign appear	(48)
	VOD	VOD decreased	(29, 49)
Stroke recurrence	Angle of branching of the small retinal arteries	Angle of branching of the small retinal arteries decreased	(51)
	MAD, MVD	MAD ≥ 74.14 μm; MVD ≥ 83.91 μm	(52)
	Fundus arteriosclerosis	positive correlation with fundus arteriosclerosis	(53)
	AVR	AVR decreased	(54)

FD, fractal dimension; FAZ, foveal avascular zone; RNFL, retinal nerve fiber layer; CRV, central retinal vein; CRA, central retinal artery; VD, vascular density; GCIPL, ganglion cell-inner plexiform layer; VOD, vascular oriented distribution; SVP, superficial vascular plexus; MAD, mean arterial diameter; MVD, mean venous diameter; AVR, arteriovenous ratio.

## 3 Application in patients with cerebral small vessel disease

CSVD is a multifaceted clinical syndrome driven by small vessel lesions in the brain. Typical MRI findings comprise recent small subcortical infarcts (RSSI), lacunae, white matter hyperintensities (WMHs), ePVS, and Cerebral microbleeds (CMBs). The onset of cerebral small-vessel disease is insidious and progressive, with a substantial lag between imaging evidence and clinical symptoms. Notably, several studies have shown that fundus lesion signs are correlated with CVSD development. This section is summarized in Table 2.

**Table 2:** Summary of retinal imaging biomarkers associated with cerebral small vessel disease.

CSVD	Related Retinal Imaging Markers	Relevance	Reference
RSSI	Retinal vascular distribution; Small vessel density; RNFL;	Sparse vascular distribution; Small vessel density decreased; RNFL decreased;	55
Lacunae	VD in the paracentral recess region of superficial capillary network	VD in the paracentral recess region of superficial capillary network decreased;	64, 66
WMHs	Artery FD; Venous curvature; Retinal curvature of the SVC; Arteriovenous dilatation;	Artery FD decreased; Venous curvature increased; Retinal curvature of the SVC increased; Arteriovenous dilatation decreased;	71
CMBs	Retinal artery; Retinal vein; Artery FD; AVR; Retinal vascular signs;	Arterial caliber stenosis; Venous caliber increased; Artery FD decreased; AVR increased; Retinal vascular signs≥2;	76 80 76, 77
ePVS	Retinal small artery; Retinal small vein; The central retinal AVR;	Arterial caliber stenosis; Venous caliber increased; The central retinal AVR decreased;	82 82 83
Total MRI burden of CSVD	OA calibre; blood flow velocity; VD;	Negatively correlated with OA calibre and blood flow velocity; VD decreased;	13, 15
CADASIL	Outer diameter of retinal arteries; Arterial wall; VD in the superficial retinal plexus of the macula;	Outer diameter of retinal arteries increased; Arterial wall thicken; VD in the superficial retinal plexus of the macula decreased;	5 5 32, 86

SVC, superficial vascular complex; OA, ophthalmic artery; CADASIL, Cerebral autosomal dominant arteriopathy with subcortical infarcts and leukoencephalopathy.

### 3.1 Recent subcortical small infarction

Recent subcortical small infarction (RSSI) is a recent infarction in the area supplied by a single perforating artery and a prevalent cause of acute IS. Although most clinical symptoms of RSSI are mild and easily overlooked, it is characterized by a high rate of recurrence, leading to an increased risk of disability and cognitive dysfunction (55). Kwapong et al. assessed retinal microvessels, choroidal capillaries, and choroidal thickness in patients with RSSI using SS-OCT and swept-source optical coherence tomography angiography (SS-OCTA) and found that retinal microvessels and choroidal are significantly thinner in RSSI patients than in controls (17). SS-OCT and SS-OCTA are new techniques for retinal imaging that have a longer wavelength, allow visualization of deeper structures, and enable very fast scanning and acquisition of three-dimensional images. Cao et al. also studied capillary imaging of the macula and optic nerve papilla in 40 RSSI patients and 46 healthy controls and found that the microvessel density of the superficial retinal capillary plexus (SRCP) (*p* = 0.001) and the deep retinal capillary plexus (DRCP) (*p* = 0.003) are significantly lower in the RSSI patients than in the healthy controls. They also showed that the SRCP density is significantly correlated with DRCP density (OR = 0.381, *p* = 0.042) (56). The retinal microvascular system spans from the RNFL to the external plexiform layer and the external nuclear layer and is composed of the SVC and the deep vascular complex (DVC) (57). Blood flows out of the SRCP into the deep veins through the DRCP. The DRCP forms the end of the macular capillary plexus, a region of thin blood vessels with low levels of oxygenation. SRCP-induced injuries are highly susceptible to damage or hypoxia-ischemia in the DRCP. Furthermore, RSSI patients have a reduced density of radial peripapillary capillar (RPC) and a significant reduction in the thickness of the pRNFL. Notably, the reduced density of radial peripapillary capillar (RPC) is significantly correlated with significant reduction in the thickness of the pRNFL. RPC is located around the optic papillae and constitutes a distinctive vascular network within the pRNFL. RPC is also associated with the metabolism of the RNFL and the ganglion cell layer (GCL) (58). RPC is more sensitive to lesions occurring in the optic papilla and macula due to its capillary narrowing (59). Changes in and around the retina reflect changes in the brain. Microcirculatory changes in the brain lead to changes in the optic microvasculature. Moreover, retinal vascular changes precede significant brain changes observed in neuroimaging (60). Therefore, effective markers and tools are useful for early screening of microangiopathy in the brain.

### 3.2 Lacunae

Lacunae are often distributed in the subcortex of the cerebrum as a round or ovoid cavity. Lacunar lesions range from 3 to 15 mm and are filled with fluid similar to the MRI signal of cerebrospinal fluid. Notably, the fluid-attenuated inversion recovery (FLAIR) sequence exhibits a low signal and a high signal around the lesion (1), consistent with acute deep cerebral infarction or hemorrhage in the area supplied by perforating arteries. The healing results in the formation of lacunar lesions (61). Notably, an increase in the number of lacunae is associated with the development of stroke and cognitive impairment, while the formation of lacunae is associated with disruption of the blood–brain barrier, deposition of fibrin in damaged blood vessels, and thickening of the wall in response to inflammatory reactions, eventually leading to occlusion of the lumen (62). The blood-retinal barrier has a similar structure (endothelial cells, pericytes, basement membrane, and surrounding neuroglia) and function (autoregulation et al.) as the blood–brain barrier (63). Unlike blood-retinal barrier, the disruption of the blood–brain barrier is difficult to detect in the early stages of the disease (48). Fu et al. demonstrated that patients with lacunar development are slightly associated with vascular signs of SRCP related to deep retinal capillaries (64). Furthermore, patients with lacunae are associated with low parafoveal capillary density (*p* < 0.001), low pericentral recess capillary density (*p* = 0.015), low RPC network density (*p* = 0.004), and thin RNFL (*p* = 0.04) in SRCP compared with controls without lacunae. Besides, comprehensive multifactorial analysis has shown that reduced VD in the paracentral recess region of the superficial capillary network (OR = 0.889, 95% CI 0.817–0.967, *p* = 0.006) is independently associated with luminal because there are two distinct vascular circulatory systems within the retina (superficial retina and deeper retina). The superficial retina is supplied by the CRA, while the deeper retina is supplied by the ciliary artery. Unlike the ciliary artery, the CRA is a terminal vessel, indicating that the ciliary artery transports more blood than the retina (65). Therefore, the deeper retina with greater blood supply is less affected by ischemia and hypoxia than the superficial layers, explaining the lack of significant correlation between deep VD and the traps. In contrast, the vascular density next to the central recess is lower than the surrounding vascular density, indicating that the retina near the central recess may also be more susceptible. Contrary to our findings, a previous study by Wang et al. reported no correlation between superficial capillary network VD and lacunae count. While both macular and peripapillary SRCP and RPC were reduced in patients compared to controls, only the former correlated with WMH after adjusting for confounding factors (66). This may be ascribed to the fact that most luminal lesions result from acute ischemic infarction (67). Whereas WMH is caused by chronic ischemic injury, the two are different forms of CSVD with different time courses and risk factors. Retinal microvascular changes may be more reflective of chronic ischemic injury to the cerebral microvasculature. Other mechanisms of CSVD, such as inflammation and endothelial dysfunction, may also be involved in the process of retinal microvascular changes. Nonetheless, more studies should elucidate the mechanisms of these microvascular changes.

### 3.3 White matter hyperintensities

WMHs are the most common subtype of CSVD, with slow and insidious disease progression and an elusive pathogenesis (68). Previous studies have shown that severe WMHs may increase the risk of vascular dementia and Alzheimer’s disease (69, 70). Therefore, early screening and diagnosis of cerebral WMHs may reduce the risk of developing CSVD. Furthermore, different retinal markers are correlated with the site of cerebral WMHs generation. A decrease in retinal arteriolar FD and an increase in venous curvature are correlated with an increase in the high signal volume of the deep cerebral white matter. Dual-specificity analysis suggested a common etiology for retinal vein curvature and deep white matter high-intensity volume. Notably, an increased volume of periventricular white matter high signal is associated with increased retinal vein diameter (71). A recent study using SS-OCTA and 3 T-MRI brain scans in normal aging adults (age ≥ 50 years) showed similar results (72). In that study, the SVC and DVC of the retina were evaluated using microvascular tortuosity (VT), and results found that deep brain WMHs are correlated with retinal curvature of the SVC (*p* = 0.027). Increased vascular tortuosity in the retina is caused by vascular wall dysfunction, tissue hypoxia, blood-retinal barrier disturbances, and blood flow disturbances. Previous studies have also shown that a similar mechanism causes high signal of white matter in the brain, which increases retinal ischemia. The body compensates for this effect through cerebral blood flow, resulting in a relative decrease in blood flow within the brain tissue, making it more susceptible to WMHs (73). Dilatation of small retinal veins, which indicates diffuse ischemia of the retina, also reflects the abnormalities of small cerebral veins. Thickening of the cerebral vein walls and vascular obstruction increases the venous pressure, dilatation of the small veins, and disruption of the blood–brain barrier. This causes ischemic stress in the brain tissues, impaired clearance of cellular metabolites from deep white matter, and WMHs. CRVE and CRAE are also risk factors for the development of WMHs in different brain regions. Increased CRVE is associated with the development of deep WMHs, whereas decreased CRAE is associated with the development of paraventricular WMHs (74). Therefore, there may be different pathogenesis and different retinal imaging markers for WMHs in different brain regions, providing a new way to screen the occurrence of WMHs in different brain regions.

Dinther et al. showed that overall extracerebral microvascular dysfunction (eMVD) can be used to assess plasma biomarkers with scintigraphic light-induced dilatation response of small retinal arteries and veins, proteinuria and glomerular filtration rate (renal), heat-induced cutaneous congestion (dermal), and endothelial dysfunction. They also showed that composite score is associated with WMH volume (75). Flicker light-induced retinal vasodilation is determined by continuous measurement of the dilation of small arteries and veins in response to flicker light using the Dynamic Vessel Analyzer (DVA). Retinal blood vessels dilate in response to flicker light stimulation through autoregulation under normal conditions. However, vasoregulation is attenuated in cases of retinal microvascular disorders, which can also be used to explore WMHs condition in the brain through retinal markers. This method is a more direct measure of retinal vascular function than markers, such as vessel diameter and density. Besides, the few literatures on both markers mostly focused on the relationship between retinal vascular geometry, microvessel density, and retinal vessel diameter with WMHs (10, 18). Therefore, future experiments should further validate the findings.

### 3.4 Cerebral microbleeds

Generally, CMBs is defined as the deposition of ferrous hemosiderin due to hemorrhage from tiny vascular lesions in the brain. This condition leads to cognitive impairment, dementia, or stroke events. A previous Reykjavik cohort study found that retinal microvascular features and fundus simple digit-like atrophy may induced by deep and lobar CMBs (76). This study demonstrated that the development of focal small retinal arteriolar stenosis (OR = 1.91, 95% CI 1.08–3.39) and arteriovenous cuts (OR = 1.96, 95% CI 1.36–2.84) was significantly associated with the formation of new CMB in the deep brain, whereas purely fundus maplike atrophy increased the risk of CMBs formation in lobar regions (OR = 2.59, 95% CI 1.01–6.65). Evidence from prior studies suggests that having ≥2 concurrent retinal vascular signs (e.g., focal small artery stenosis, arteriovenous crossover sign, retinal speckle hemorrhage, microaneurysms, soft exudates) increases the incidence of deep brain microhemorrhages by a factor of 3 but is not associated with occurrence of microhemorrhages in lobar areas. A recent investigation by Anisetti et al. reported the existence of cerebral amyloid angiopathy (CAA) in patients diagnosed with age-related macular degeneration (AMD). CAA is prevalent and strongly linked to lobar, but not deep CMBs. Patients with advanced AMD exhibited a 2.83-fold increased risk of possible CAA and a 1.85-fold elevated risk of lobar CMBs compared to AMD-free controls (77). These findings suggest that the occurrence CMBs in different locations may be driven by different mechanisms, which leads to the formation of lesions in the ocular retina. Considering similarities in anatomical and physiological features, such as the blood-retinal barrier and autoregulation of blood pressure, changes in retinal vessel diameters may reflect similar changes in small arteries and veins of the brain. Besides the retinal signs discussed above, several other studies have reported that smaller central retinal caliber, larger caliber of small retinal veins, and smaller FD of small arteries are also associated with the occurrence of multiple CMBs (78, 79). In contrast, a recent study investigating the relationship between retinal AVR and CMBs found that patients with CMBs had a lower AVR compared to healthy controls (*p* < 0.023), and AVR was negatively correlated with the number of CMBs (ß = −0.063, *p* = 0.035) (80). In addition, Alber et al. examined the retinal imaging structures and angiographic changes in patients with CAA and found a trend-level correlation between the number and surface area of retinal microhemorrhages and the number of cerebral cortical microhemorrhages in patients with CAA relative to health controls (81). Although prior studies have revealed the mechanisms underlying the occurrence of microhemorrhages in different sites to some extent, future investigations are needed to identify markers such as retinal vascular signs for specific microhemorrhage sites in the brain, which can help to predict the possible sites of new CMBs formation.

### 3.5 Enlarged perivascular space

The perivascular space (PVS) is a fluid-filled gap that surrounds small blood vessels within the brain. Hernandez-diaz et al. reported a correlation between retinopathy severity and the prevalence of ePVS, with higher retinopathy grades (II–III) associated with increased ePVS incidence (78). In contrast, Mutlu et al. explored ePVS location and its association with retinal arteriolar and venular caliber within specific brain regions: the centrum semiovale, basal ganglia, and hippocampus. The results showed that narrowing of the caliber of small arteries and widening of the caliber of small veins were correlated with the development of ePVS in the semiovale center and hippocampus, but not with the occurrence of ePVS in the basal ganglia (82). However, a different study on the CRA diameter and vein ratio found that a smaller central retinal AVR (ß = −3.974, 95% CI −5.548 to −2.400, *p* = 0.012), correlated with a severer ePVS (83). The above investigations examined the correlation between retinal vascular changes and ePVS. Given that the perivascular space drains interstitial and cerebrospinal fluid into the subarachnoid space and eventually into the cervical lymph nodes, the vascular lesions of this transport causes variations in hemodynamic pressures, which may manifest in the vascular caliber changes leading to arterial atrophy or venous dilatation and enlargement of the perivascular gap. Further studies are warranted to explore the exact mechanisms.

### 3.6 Total MRI burden of CSVD

In recent years, the “total MRI burden of CSVD” has been proposed is a new indicator. Specifically, it is a scale that comprehensively evaluates the total imaging burden of CSVD by incorporating the four most common imaging manifestations, namely CSVD, lacunar, WMHs, CMBs, and ePVS, which are scored on a scale of 0–4 (84). The correlation between ophthalmic artery (OA) morphology and hemodynamics and the total CSVD score in patients with CSVD has been recently investigated. The results indicated that the total score was negatively correlated with the OA diameter and blood flow velocity (both *p* < 0.05), and the total CSVD score was independently correlated with blood flow velocity in the OA after correction for potential confounders (*β* = −0.202, *p* = 0.005) (15). It was found that patients with a total CSVD score of 4 had significantly lower arterial diameters than healthy controls, and this was ascribed to the pathologic changes in the small arteries in the brain of CSVD patients. Arterial lesions contain hyaline and proliferative atherosclerosis of the small arteries, which may increase the midlumen/lumen ratio. Vessel wall damage triggers the accumulation and deposition of abnormal proteins and collagen, leading to small cerebral vessel thickening and lumen narrowing. A similar process might contribute to reduced OA diameter in CSVD. Given that the CRA branches from the OA, OA lesions can significantly impact retinal vessel vascularity and blood flow. Hiroki et al. found that end-diastolic flow velocity and mean flow velocity of the CRA in patients with CSVD were significantly lower compared with that of healthy controls, and the CRA flow velocities correlated with the score evaluation of CSVD (13). Lee et al. found that decreased retinal capillary density in patients with CSVD was negatively correlated with the total CSVD score (9). Shu et al. suggested that retinal microvascular abnormalities can predict severe total CSVD load (85). This indicates that the fundus vascular abnormalities can reflect the severity of total CSVD burden and may be a robust biomarker for screening and treating CSVD in the future.

### 3.7 Cerebral autosomal dominant arteriopathy with subcortical infarcts and leukoencephalopathy

Cerebral autosomal dominant arteriopathy with subcortical infarcts and leukoencephalopathy (CADASIL) is an inherited cerebral small-vessel disease caused by mutations in the NOTCH3 gene on chromosome 19 (66). Fang et al. detected fundus lesions in patients with CADASIL and reported that the outer diameter of retinal arteries was increased and arterial walls were thickened in CADASIL patients compared with healthy controls (5). A recent study by Lin et al. investigated vascular density in the macular region of the eyes of CADASIL patients (divided into stroke and non-stroke groups) and healthy controls. They found that compared with healthy subjects, stroke patients exhibited significantly lower VD in the superficial retinal plexus of the macula, and that VD and intraretinal thickness were positively correlated with gait speed and negatively correlated with the number of cavities in the brain, but there was no significant difference in VD between the total CADASIL group and the control group (86). These findings diverge from those of Nelis et al., who reported significantly reduced macular vascular density in CADASIL patients compared to healthy controls (32). This difference may be due to the inclusion of different subgroups and variations in the disease course. In the study, they subdivided patients into stroke versus non-stroke subgroups, whereas in the study by Nelis et al. this patient classification was not performed and the reduction in vascular density was a slower process that could serve as a marker of the severity of CADASIL in its later stages, but not an early manifestation. Moreover, both studies lacked a standardized disease severity assessment for CADASIL patients and included relatively small sample sizes. These limitations could contribute to the observed discrepancies in findings.

## 4 Application in vascular cognitive impairment

Clinically, VCI comprises of syndromes ranging from mild cognitive impairment to dementia triggered by risk factors of cerebrovascular disease. It is caused by vasogenic cognitive impairments, from mild to severe. Available evidence suggests that cerebrovascular lesions contributes to cognitive impairment and dementia and exert a synergistic or superimposed effect with neurodegenerative lesions (87). Past the AGES-Reykjavik study reported that retinopathy was associated with Vascular dementia (VaD) and that patients with retinopathy and cerebral microinfarcts showed increased risk of dementia (88). Vascular dementia is relatively rare and often co-occurs or interacts with neurodegenerative conditions like Alzheimer’s disease, complicating its study. Marquié et al. divided 672 participants into five groups as follows: cognitively unimpaired (CU), mild cognitive impairment due to Alzheimer’s disease (MCI-AD), MCI due to cerebrovascular pathology (MCI-Va), probable Alzheimer’s disease dementia (ADD), and VaD. They compared differences in superficial plexiform macular VD in the four quadrants (superior, nasal, inferior and temporal) in superficial plexiform macular VD (89). The results showed differences between the two MCI groups (MCI-AD and MCI-Va), with significantly higher VD in the temporal quadrant of MCI-AD compared to participants with no cognitive abnormalities (49.05 ± 4.91 vs. 47.27 ± 4.17, *p* = 0.02), and significantly lower VD in the inferior and middle quadrants of MCI-Va compared to normal controls (48.70 ± 6.57 vs. 51.27 ± 6.39, *p* = 0.02). These findings indicate that retinal vascular density may serve as an early biomarker to differentiate between Alzheimer’s disease and vascular etiologies of cognitive decline, with potential diagnostic utility diminishing in later disease stages. In contrast, Wang et al. found that the VD of the SRCP in the macula was associated with cognitive functions, especially global cognition, memory function, attentional function, information processing, and executive function. This implies that patients with low vascular density in the retina are more likely to have poorer overall cognitive performance and significant cognitive deficits in individual domains (66). Previous studies have linked reduced retinal blood perfusion and enlarged foveal avascular zone (FAZ) to cognitive decline (83, 90, 91). Elsewhere, Abdolahi et al. investigated retinal perfusion metrics using a new cognitive assessment, the NIH Toolbox Cognitive Test (NIHTB-CB), to monitor changes in cognitive function in people included in the experiment, and the results suggested that lower retinal capillary perfusion was associated with poorer speed of information processing (*β* = 0.42, 95%CI 0.19–0.65, *p* < 0.001,) and fluid perception (β = 0.28, 95% CI 0.04, −0.52, *p* = 0.022). Researchers have proposed that altered retinal perfusion may be a biomarker for early cognitive changes during the development of cerebrovascular disease (92). A recent studies have also found an association between retinal RNFL thickness and cognitive changes. Cui et al. studied the cognitive status and retinal imaging results of 132 patients with RSSI and found a significant correlation between Montreal Cognitive Assessment (MoCA) scores and RNFL in patients with RSSI, suggesting that retinal imaging marker measurements may be a useful tool for identifying cognitive deterioration in these individuals (93). The above results suggest that retinal imaging markers are useful indicators for screening individuals at high risk for VCI, and can facilitate early detection, monitoring disease progression, and response to therapy. This section is summarized in Table 3.

**Table 3:** Summary of retinal imaging biomarkers associated with VCI.

VCI	Related retinal imaging markers	Relevance	Reference
VCI	VD in the superficial retinal plexus of the macula	VD in the superficial retinal plexus of the macula decreased	(66, 89)
	Retinal blood perfusion	Retinal blood perfusion decreased	(92)
	FAZ area	FAZ area increased	(83, 90, 91)
	RNFL	RNFL decreased	(93)

VCI, Vascular cognitive impairment; RNFL, retinal nerve fiber layer.

## 5 Conclusion

**Figure 2:**
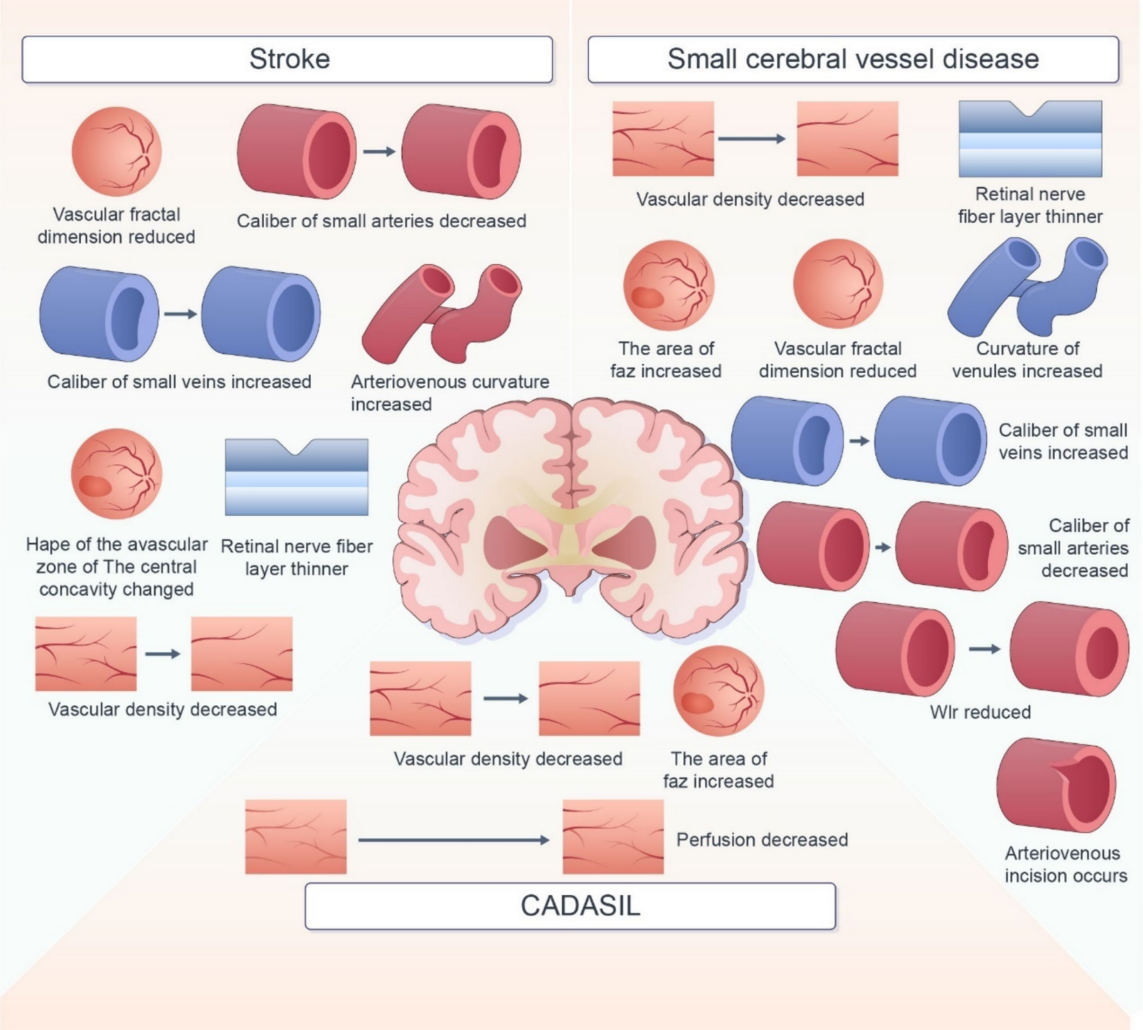
Summarized retinal imaging biomarker changes in different cerebrovascular diseases.

In this review, we summarized the retinal imaging markers associated with various cerebrovascular diseases (Figure 2). Retinal imaging, as a non-invasive and easily accessible examination tool, overcomes the limitations of traditional imaging screening tools for cerebrovascular disease. Several biomarkers with potential to predict the occurrence, development, and prognosis of cerebrovascular diseases have been reported. But The current use of retinal imaging biomarkers in the diagnosis of cerebrovascular disease is still affected by several factors. First, in terms of technical imaging, the current imaging technology is not able to detect some early or small lesions. It is not able to describe the morphology of more complex markers accurately, which may affect the identification of markers. As for markers, many retinal imaging biomarkers are not specific to cerebrovascular disease. The impact of demographic factors such as age, gender, and region, as well as comorbid factors such as hypertension and diabetes, also need to be considered. These increase the difficulty of judging marker specificity. Moreover, the Pathology, physiological mechanisms between retinal markers and cerebrovascular disease is not yet completely clear. There is also no uniform standard for diagnosis. Many studies have used different markers and diagnostic thresholds, which makes it difficult to determine a clear diagnostic basis for retinal imaging biomarkers in practical application. Therefore, there are still some limitations in the diagnosis of cerebrovascular diseases with retinal imaging biomarkers. Thus, development of imaging techniques, additional investigations using uniform and standardized assessment methods are warranted to assess the correlation between retinal signs and cerebrovascular lesions. Rapid advancements in science and technology have led to the development of innovative retinal imaging techniques, such as OCT, OCTA, SS-OCT, and infrared imaging. These technologies enable detailed visualization and quantitative assessment of fundus and vascular structures, including retinal thickness and blood flow density. In the future, techniques such as high-resolution imaging, noninvasive detection techniques, and automated analysis by intelligent computers should be employed to improve the accuracy and applicability of retinal imaging biomarkers in the diagnosis and prevention of cerebrovascular disease.
