# Corrigendum: Advances in retinal imaging biomarkers for the diagnosis of cerebrovascular disease

**DOI:** 10.3389/fneur.2024.1529001

**Published:** 2024-12-02

**Authors:** Yier Zhang, Ting Zhao, Ling Ye, Sicheng Yan, Wuyue Shentu, Qilun Lai, Song Qiao

**Affiliations:** ^1^The Second Clinical Medical College, Zhejiang Chinese Medicine University, Hangzhou, Zhejiang, China; ^2^Department of Neurology, Zhejiang Hospital, Hangzhou, Zhejiang, China; ^3^Department of Geriatrics, Jinhua Fifth Hospital, Jinhua, Zhejiang, China

**Keywords:** cerebrovascular disease, Retina, biomarkers, Cerebral microcirculation, brain function

In the published article, there was an error regarding the affiliation(s) for Song Qiao. In the original article, the author's affiliation(s) were 1,2.

Their correct affiliation is: ^2^ Department of Neurology, Zhejiang Hospital, Hangzhou, Zhejiang, China.

The authors apologize for this error and state that this does not change the scientific conclusions of the article in any way. The original article has been updated.

